# Simultaneous Determination of Original, Degraded Ginsenosides and Aglycones by Ultra High Performance Liquid Chromatography Coupled with Quadrupole Time-of-Flight Mass Spectrometry for Quantitative Evaluation of Du-Shen-Tang, the Decoction of Ginseng

**DOI:** 10.3390/molecules19044083

**Published:** 2014-04-02

**Authors:** Shan-Shan Zhou, Jin-Di Xu, He Zhu, Hong Shen, Jun Xu, Qian Mao, Song-Lin Li, Ru Yan

**Affiliations:** 1 Department of Pharmaceutical Analysis and Metabolomics, Jiangsu Province Academy of Traditional Chinese Medicine and Jiangsu Branch of China Academy of Chinese Medical Sciences, Nanjing 210028, China; 2 College of Pharmacy, Nanjing University of Chinese Medicine, Nanjing 210046, China; 3 State Key Laboratory of Quality Research in Chinese Medicine, Institute of Chinese Medical Sciences, University of Macau, Macao, China

**Keywords:** UHPLC-QTOF-MS, ginseng, degraded ginsenosides, quantification, Du-Shen-Tang, decoction

## Abstract

In the present study, an ultra-high performance liquid chromatography coupled with quadrupole time-of-flight mass spectrometry (UHPLC-QTOF-MS) method for simultaneous determination of eleven original, fourteen degraded ginsenosides and five aglycones was developed and validated to quantitatively evaluate the transformation of ginsenosides during preparation of Du-Shen-Tang, the decoction of ginseng. Both positive and negative modes as well as the step wave ion transfer optics technology were used to increase the detection sensitivity of QTOF-MS. The extracting ion mode based on the quasi-molecular ions, molecular ions and fragment ions characteristic to each analyte was used to increase the selectivity for quantitative analysis. Under the optimized UHPLC and QTOF-MS conditions, the 30 analytes with different polarities were separated **(**except for Re and Rg_1_**)** within 26 min. The developed method was applied for the quantitative comparison of Du-Shen-Tang and its raw materials derived from Asian ginseng (ASG) and American ginseng (AMG), respectively. It was found that the contents of the original ginsenosides decreased from 26,053.09 to 19,393.29 μg/g or 45,027.72 to 41,865.39 μg/g, whereas the degraded ginsenosides and aglycones increased from 159.72 to 685.37 μg/g or 676.54 to 1,502.26 μg/g in Du-Shen-Tang samples of ASG or AMG when compared with their raw materials, indicating that decocting could dramatically increase the proportion of the less polar degraded ginsenosides in Du-Shen-Tang. Whether these changed proportions of different polar ginsenosides could affect the bioactivities of the decoctions and their raw materials derived from ASG and AMG deserves further investigation.

## 1. Introduction

Ginseng, the root and rhizome of *Panax ginseng* (Asian ginseng, ASG) or *P. quinquefolius* (American ginseng, AMG), has been used as a tonic and panacea to promote longevity in traditional Chinese medicine (TCM) for thousands of years [1,2,3]. Du-Shen-Tang, the decoction of ginseng, which was firstly documented in “*Shi Yao Shen Shu*” (an ancient monograph on ten magic herbs published about 660 years ago in China Yuan dynasty), has been used to “invigorate *qi* for relieving desertion”, and was regarded as a paragon of emergency treatment in TCM practice [4]. Du-Shen-Tang is currently prescribed to treat cardiogenic shock [5], expansion of myocardium [6] and for stimulation of parturition [7]. 

Modern phytochemical and pharmacological studies revealed that ginsenosides which were generally classified as protopanaxadiol (PPD), protopanaxtriol (PPT) and oleanolic acid (OCO) types based on their aglycone moieties, are bioactive components that may contribute mainly to the efficacy of ginseng [2]. Accumulated data suggest that the less polar ginsenosides are more potent and show higher bioavailability than those with relatively higher polarity [8,9,10,11,12]. Our previous study found that during preparation of Du-Shen-Tang, the original ginsenosides (with relatively higher polarity) such as Rb_1_, Rb_2_, Rb_3_, Rc, Rd, Rg_1_, Re, Rf, *etc.* could be degraded to generate ginsenosides with relatively less polarity, such as Rg_3_, Rh_2_, Rh_1_, Rg_2_, *etc.*, through hydrolysis, dehydration and decarboxylation, *etc.*, resulting in the chemical profiles of Du-Shen-Tang different from those of respective raw materials [13]. In order to standardize the preparation procedure and evaluate the holistic quality of Du-Shen-Tang, the contents of original ginsenosides as well as those of the degraded products should be quantified. 

Many methods have been reported for quantification of ginsenosides in ginseng raw materials, such as high performance liquid chromatography-ultraviolet detection (HPLC-UV) [14], ultra performance liquid chromatography-ultraviolet detection (UPLC-UV) [15], high performance liquid chromatography-evaporative light scattering detection (HPLC-ELSD) [16,17,18], and high performance liquid chromatography-fluorescence detection (HPLC-FLD) [19,20], *etc.* However, the UV detection offers low sensitivity due to poor UV absorption of ginsenosides; ELSD provides better sensitivity than UV for ginsenosides detection, but online chemical structure information is not available for analytes identification or confirmation; and FLD requires tedious fluorogenic derivatization of ginsenosides before analysis [20]. Recently, liquid chromatography coupled with triple quadrupole mass spectrometry (LC-TQ-MS) or quadrupole/time of flight mass spectrometry (LC-QTOF-MS) was widely employed for qualitative and quantitative analysis of medicinal herbs or complex prescriptions [21,22,23]. Compared with TQ-MS, QTOF-MS provides accurate mass measurement, high resolution and selectivity, which makes LC-QTOF-MS attractive for simultaneously qualitative and quantitative analysis of complicated analytes in medicinal herbs or complex prescriptions [24,25]. Recently, a LC-QTOF-MS method was reported for simultaneous quantification of 15 ginsenosides in biological samples within a 35-min run [26]. However, aglycones were not considered in all these studies. More recently, a rapid resolution liquid chromatography coupled with tandem mass spectrometry (RRLC-TQ-MS) method was reported for the quality evaluation of Du-Shen-Tang, but only seven original ginsenosides were quantified [27].

Ultra-high performance liquid chromatography (UHPLC) is an advanced chromatographic technique, holding enhanced retention time reproducibility, high chromatographic resolution, improved sensitivity and shorter operation time [28]. In present study, an UHPLC-QTOF-MS/MS method for simultaneous determination of 30 analytes including original, degraded ginsenosides and aglycones was developed and validated, and applied to compare the contents of these analytes in Du-Shen-Tang with those present in its raw materials derived from ASG and AMG.

## 2. Results and Discussion

### 2.1. Optimization of Chromatographic Conditions

**Figure 1:**
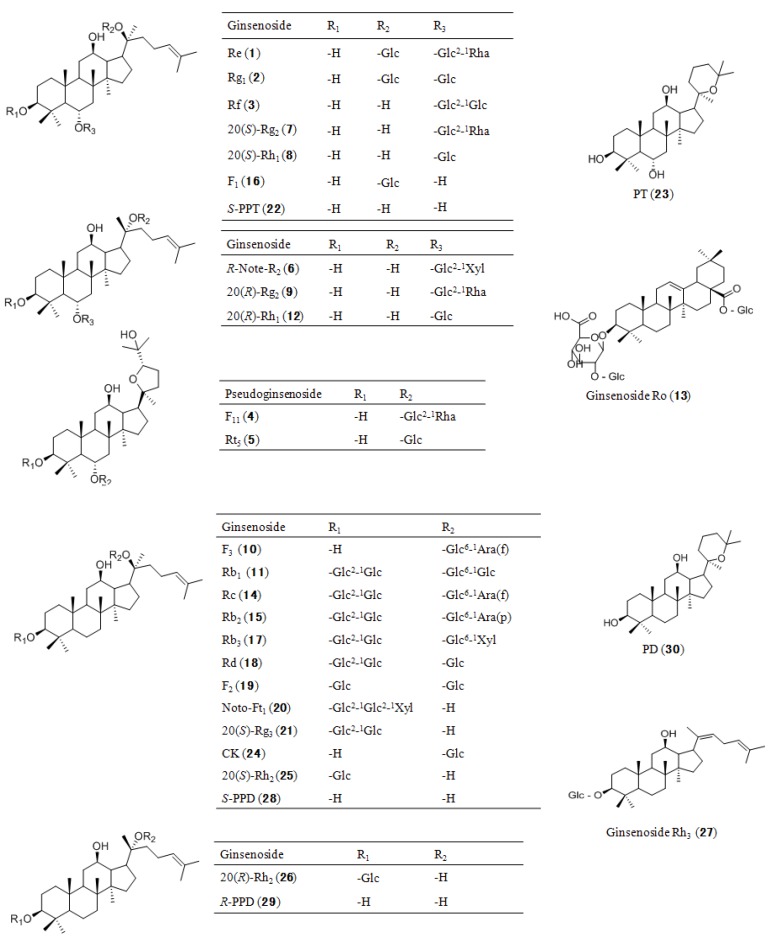
Chemical structures of 30 ginsenosides and aglycones. Glc, β-d-glucopyranosyl; Ara(p), α-l-arabinopyranosyl; Ara(f), α-l-arabinofuranosyl; Rha, α-l-rhamnopyranosyl; Xyl, β-d-xylopyranosyl.

**Figure 2:**
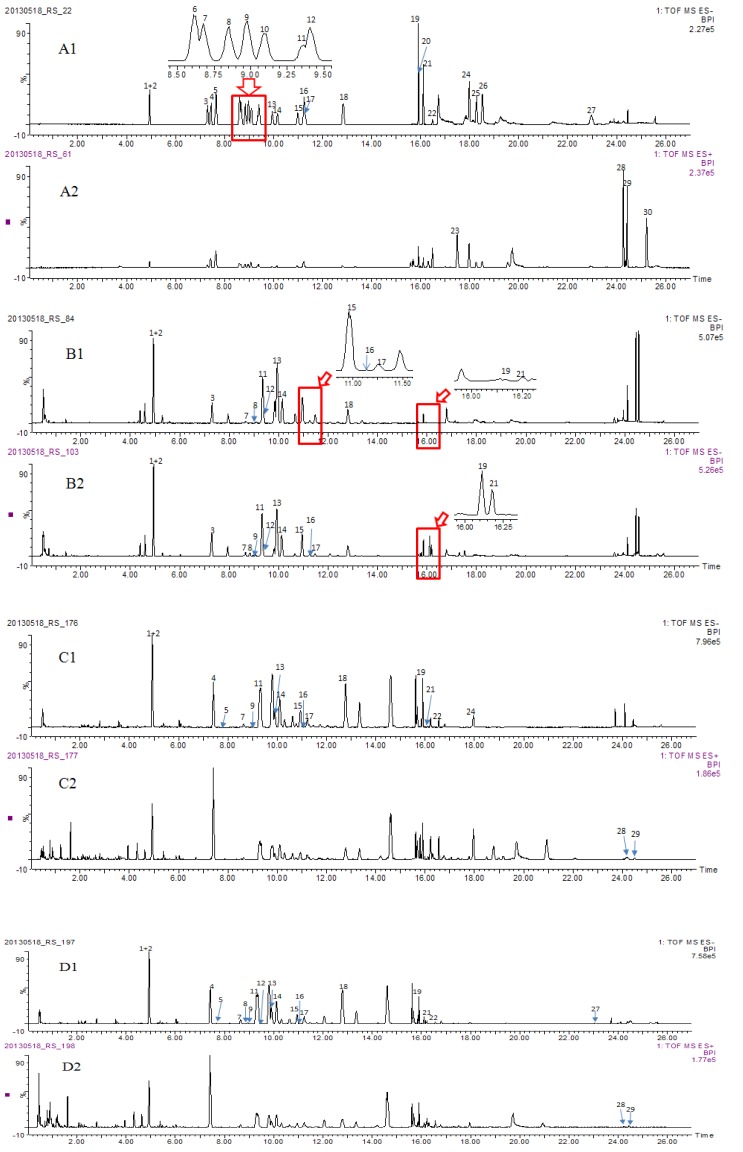
BPI chromatograms of reference compounds, the methanol extracts and the decoctions (Du-Shen-Tang) of ASG and AMG by UHPLC–QTOF–MS/MS analysis. A: Chromatograms of 30 reference compounds in negative (**A1**) and positive (**A2**) ion mode; B: Chromatograms of the methanol extract (**B1**) and the decoction (**B2**) of ASG in negative ion mode; C: Chromatograms of the methanol extract of AMG in negative ion mode (**C1**) and positive ion mode (**C2**); D: Chromatograms of the decoction of AMG in negative ion mode (**D1**) and positive ion mode (**D2**). Compound codes are the same as those in Figure 1.

To develop a method for simultaneously quantifying original ginsenosides and degraded products that co-existed in Du-Shen-Tang, the chromatographic conditions were optimized based on our previous studies [13,29]. Thirty main components of ginseng including eleven original ginsenosides (with relatively higher polarity), fourteen degraded ginsenosides and five aglycones (Figure 1) which were not detected in our previous studies (with relatively less polarity) were chosen as marker compounds to optimize the conditions. Three UHPLC columns, *i.e.*, ACQUITY HSS T3 (100 mm × 2.1 mm, 1.8 μm), ACQUITY BEH (100 mm × 2.1 mm, 1.7 μm) and ACQUITY CSH (100 mm × 2.1 mm, 1.7 μm) and different mobile phase compositions (MeOH–H_2_O, ACN–H_2_O, acidic or not) were tested. It was found that all the 30 selected analytes could be separated on ACQUITY HSS T3 (100 mm × 2.1 mm, 1.8 μm) column with better resolution within 26 min (Figure 2A1, A2) when eluted with acidic ACN and water in a gradient mode, faster than the reported LC-QTOF-MS method in which 35 min were needed for separation of mere 15 ginsensosides [26]. Therefore, the chromatographic conditions were chosen as described in Section 2.3.

### 2.2. Optimization of MS Conditions

It is well known that LC-TQ-MS with multiple reactions monitoring (MRM) mode is usually more sensitive than LC-QTOF-MS for quantitative analysis, whereas LC-QTOF-MS with accurate mass measurement is more suitable than LC-TQ-MS for qualitative analysis [25]. In present study, a Q/TOF-MS with step wave ion-transfer optics, a newly developed technology for improving quantitative sensitivity of QTOF-MS [30], was used for simultaneously qualitative and quantitative determination of different types of ginsenosides and aglycones in Du-Shen-Tang. Our preliminary study found that ginsenosides could be detected with higher sensitivity and abundant fragment ions in negative ion mode, whereas their aglycones including *S*-PPD, *R*-PPD, PT and PD could be detected with higher sensitivity and clearer mass spectra in positive ion mode. Thus both positive and negative ion modes with the optimized collision energy, cone voltage and capillary voltage were employed to monitor the target analytes of Du-Shen-Tang with higher accuracy and sensitivity.

### 2.3. Identity Confirmation and Selection of Extracting Ions for Quantification of 30 Analytes

**Figure 3:**
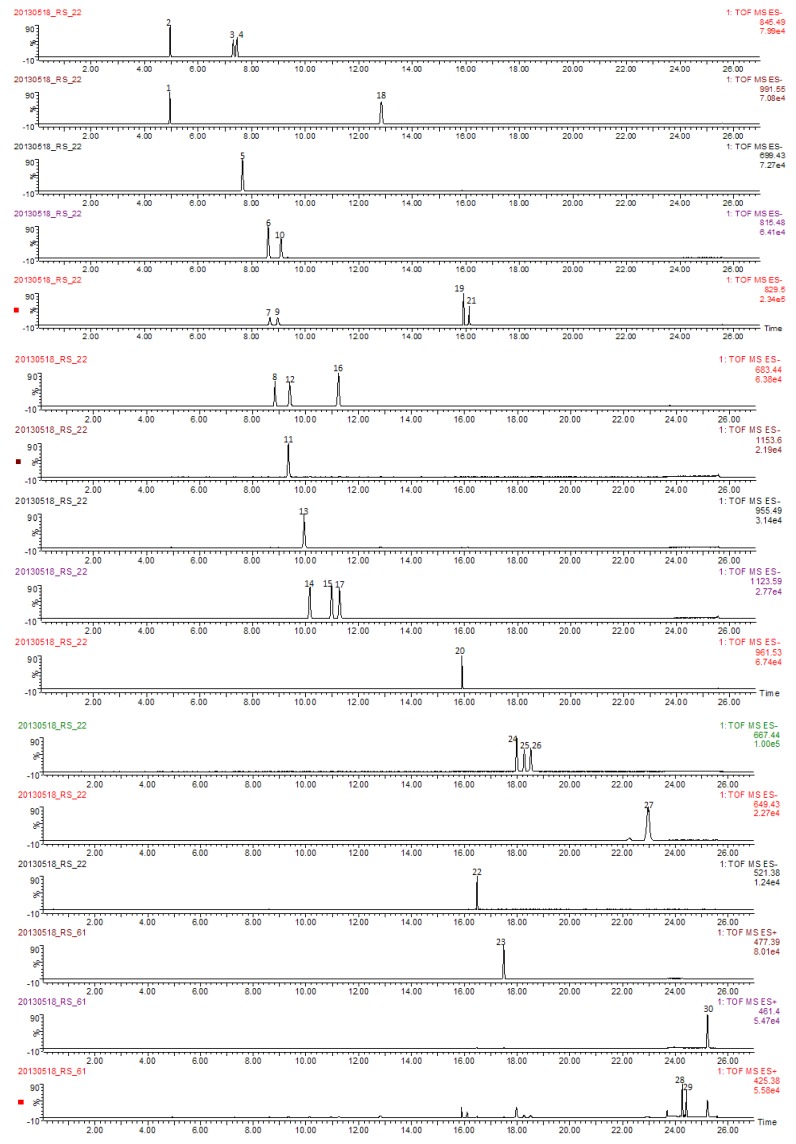
Extracted ion chromatograms of 30 analytes (6.25 μg/mL each in reference compound mixture solution. Compound codes are the same as those in Figure 1.

The identities of the peaks interested in Du-Shen-Tang were confirmed by comparing their retention times, mass spectra, accurate mass data of molecular ions and fragment ions with those of the 30 reference compounds (Table 1). Under the optimized conditions, it was found that the PPT and PPD type ginsenosides showed abundant formic acid adduct ions [M−H+HCOOH]^−^, the OCO type ginsenosides exhibited abundant deprotonated molecular ions [M−H]^−^, while the aglycones produced abundant protonated molecular ions [M+H]^+^ or dehydration ions [M+H−2H_2_O]^+^. Therefore, these ions characteristic to each analyte were selected as the extracting ions to get higher selectivity and sensitivity for quantitative analysis. The extracting ions selected for each analyte were summarized in Table 1, and the chromatograms of extracting ions for all analytes were shown in Figure 3. It was found that all analytes could be monitored with baseline separation under developed analytical conditions.

### 2.4. Method Validation

The calibration curves, regression coefficients and concentration ranges of 30 reference compounds were summarized in Table 1. It was found that all 30 analytes showed good linearity (R^2^ ≥ 0.997) within the tested concentration ranges. 

The overall LOQ and LOD of the analytes reached 0.02 ng and 0.01 ng, respectively, and the RSDs of intra- and inter-day variations were less than 8.79% and 9.46%, respectively, for 30 analytes (Table 2). As to the stability test, the RSDs of the peak areas for 30 analytes detected within 24 h were lower than 7.73% (Table 2). Furthermore, the developed method also provided acceptable accuracy with spike recovery ranging from 86.30% to 114.84% for majority of the analytes (except for Re and Rg1) (Table 2). All these results demonstrated that the established UHPLC-QTOF-MS/MS method offers adequate linearity, sensitivity, precision, accuracy, and stability for simultaneous determination of 30 components including original, degraded ginsenosides and aglycones co-existed in decoctions or raw materials of ginseng. 

It should be noticed that under the present conditions, the spike recoveries of ginsenoside Re and Rg_1_ were relatively lower (36.05%–37.72% and 44.51%–45.79%, respectively), which might be due to the ionization suppression of these two co-eluting analytes [31]. The true contents of these two analytes in ginseng raw materials and decoctions were roughly calculated with the following formula:

True values = Values calculated from calibration curves/Average recovery

**Table 1:** Retention times, extracting ions for quantification, calibration curves and ions for identity confirmation of 30 analytes.

No.	Analyte	t_R_ (min)	Extracting Ion (*m/z*)	Range (μg/mL)	Equation	R^2^	Ions for Identity Confirmation *
1	Re	4.96	991.55[M–H+HCOOH]^−^	0.39–50.00	y = 278.10x + 118.50	0.997	991.5482[M−H+HCOOH]^−^ (991.5478, 0.4)
							981.5180[M+Cl]^−^ (981.5190, −1.0)
							945.5443[M–H]^−^ (945.5423, 2.1)
							799.4845[M–H–(Rha–H2O)]^−^ (799.4844, 0.1)
							783.4921[M–H–(Glc–H2O)]^−^ (783.4895, 3.3)
							637.4335[M–H–(Rha–H2O)–(Glc–H2O)]^−^ (637.4316, 3.0)
							475.3779[M–H–(Rha–H2O)–2(Glc–H2O)]^−^ (475.3787, −1.7)
2	Rg_1_	4.97	845.49[M–H+HCOOH]^−^	0.39–50.00	y = 284.00x + 284.70	0.992	845.4905[M–H+HCOOH]^−^ (845.4899, 0.7)
							835.4617[M+Cl]^−^ (835.4611, 0.7)
							799.4845[M–H]^−^ (799.4844, 0.1)
							637.4335[M–H–(Glc–H2O)]^−^ (637.4316, 3.0)
							475.3773[M–H–2(Glc–H2O)]^−^ (475.3787, −2.9)
3	Rf	7.35	845.49[M–H+HCOOH]^−^	0.39–50.00	y = 283.00x − 39.76	0.998	845.4886[M–H+HCOOH]^−^ (845.4899, −1.5)
							835.4595[M+Cl]^−^ (835.4611, −1.9)
							799.4806[M–H]^−^ (799.4844, −4.8)
							637.4305[M–H–(Glc–H2O)]^−^ (637.4316, −1.7)
							475.3785[M–H–2(Glc–H2O)]^−^ (475.3787, −0.4)
4	Pseudo-F_11_	7.57	845.49[M–H+HCOOH]^−^	0.39–50.00	y = 242.90x + 118.00	0.995	845.4900[M–H+HCOOH]^−^ (845.4899, 0.1)
							835.4623[M+Cl]^−^ (835.4611, 1.4)
							799.4850[M–H]^−^ (799.4844, 0.8)
							653.4268[M–H–(Rha–H2O)]^−^ (653.4265, 0.5)
							635.4162[M–H–(Rha–H2O)–H2O]^−^ (635.4159, 0.5)
							491.3747[M–H–(Rha–H2O)–(Glc–H2O)]^−^ (491.3736, 2.2)
5	Pseudo-Rt_5_	7.72	699.43[M–H+HCOOH]^−^	0.39–12.50	y = 659.60x − 133.60	0.998	699.4313[M–H+HCOOH]^−^ (699.4320, −1.0)
							689.4020[M+Cl]^−^ (689.4032, −1.7)
							653.4271[M–H]^−^ (653.4265, 0.9)
							491.3746[M–H–(Glc–H2O)]^−^ (491.3736, 2.0)
6	*R*-Note-R_2_	8.68	815.48[M–H+HCOOH]^−^	0.39–12.50	y = 642.30x − 217.40	0.998	815.4788[M–H+HCOOH]^−^ (815.4793, −0.6)
							805.4504[M+Cl]^−^ (805.4505, −0.1)
							769.4731[M–H]^−^ (769.4738, −0.9)
							637.4323[M–H–(Xyl–H2O)]^−^ (637.4316, 1.1)
							475.3782 [M–H–(Xyl–H2O)–(Glc–H2O)]^−^ (475.3787, −1.1)
7	20(*S*)-Rg_2_	8.74	829.50[M–H+HCOOH]^−^	0.39–50.00	y = 414.40x − 19.21	0.999	829.4952[M–H+HCOOH]^−^ (829.4949, 0.4)
							819.4666[M+Cl]^−^ (819.4661, 0.6)
							783.4897[M–H]^−^ (783.4895, 0.3)
							637.4311[M–H–(Rha–H2O)]^−^ (637.4316, –0.8)
							475.3786[M–H–(Rha–H2O)–(Glc–H2O)]^−^ (475.3787, −0.2)
8	20(*S*)-Rh_1_	8.91	683.44[M–H+HCOOH]^−^	0.39–100.00	y = 419.50x − 80.11	0.999	683.4369[M–H+HCOOH]^−^ (683.4370, –0.1)
							673.4070[M+Cl]^−^ (673.4082, −1.8)
							637.4312[M–H]^−^ (637.4316, −0.6)
							475.3778 [M–H–(Glc–H2O)]^−^ (475.3787, −1.9)
9	20(*R*)-Rg_2_	9.04	829.50[M–H+HCOOH]^−^	0.39–100.00	y = 516.10x − 8.84	0.999	829.4946[M–H+HCOOH]^−^ (829.4949, −0.4)
							819.4650[M+Cl]^−^ (819.4661, −1.3)
							783.4904[M–H]^−^ (783.4895, 1.1)
							637.4330[M–H–(Rha–H2O)]^−^ (637.4316, 2.2)
							475.3785[M–H–(Rha–H2O)–(Glc–H2O)]^−^ (475.3787, −0.4)
10	F_3_	9.17	815.48[M–H+HCOOH]^−^	0.39–100.00	y = 353.60x + 90.34	0.999	815.4788[M–H+HCOOH]^−^ (815.4793, −0.6)
							805.4503[M+Cl]^−^ (805.4505, −0.2)
							769.4730[M–H]^−^ (769.4738, −1.0)
							637.4324[M–H–(Xyl–H2O)]^−^ (637.4316, 1.3)
							475.3792 [M–H–(Xyl–H2O)–(Glc–H2O)]^−^ (475.3787, 1.1)
11	Rb_1_	9.43	1153.60[M–H+HCOOH]^−^	0.39–100.00	y = 234.20x − 114.70	0.999	1153.6013[M–H+HCOOH]^−^ (1153.6006, 0.6)
							1143.5693[M+Cl]^−^ (1143.5718, −2.2)
							1107.5957[M–H]^−^ (1107.5951, 0.5)
							945.5427[M–H–(Glc–H_2_O)]^−^ (945.5423, 0.4)
							783.4916[M–H–2(Glc–H_2_O)]^−^ (783.4895, 2.7)
							621.4380[M–H–3(Glc–H_2_O)]^−^ (621.4366, 2.3)
12	20(*R*)-Rh_1_	9.48	683.44[M–H+HCOOH]^−^	0.39–100.00	y = 395.80x + 69.26	0.999	683.4382[M–H+HCOOH]^−^ (683.4370, 1.8)
							673.4080[M+Cl]^−^ (673.4082, −0.3)
							637.4310[M–H]^−^ (637.4316, −0.9)
							475.3799[M–H–(Glc–H_2_O)]^−^ (475.3787, 2.5)
13	Ro	10.02	955.49[M–H]^−^	0.39–100.00	y = 284.40x − 183.20	0.999	955.4890[M–H]^−^ (955.4903, −1.4)
							793.4354[M–H–(Glc–H_2_O)]^−^ (793.4374, −2.5)
14	Rc	10.25	1123.59[M–H+HCOOH]^−^	0.39–100.00	y = 257.80x − 206.90	0.998	1123.5935[M–H+HCOOH]^−^ (1123.5900, 3.1)
							1113.5659[M+Cl]^−^ (1113.5612, 4.2)
							1077.5852[M–H]^−^ (1077.5845, 0.6)
							945.5405[M–H–(Ara(f)–H_2_O)]^−^ (945.5423, −1.9)
							915.5335[M–H–(Glc–H_2_O)]^−^ (915.5317, 2.0)
							783.4894[M–H–(Ara(f)–H_2_O)–(Glc–H_2_O)]^−^ (783.4895, −0.1)
							621.4370[M–H–(Ara(f)–H_2_O)–2(Glc–H_2_O)]^−^ (621.4366, 0.6)
15	Rb_2_	11.07	1123.59[M–H+HCOOH]^−^	0.39–100.00	y = 319.20x − 281.40	0.998	1123.5908[M–H+HCOOH]^−^ (1123.5900, 0.7)
							1113.5627[M+Cl]^−^ (1113.5612, 1.3)
							1077.5858[M–H]^−^ (1077.5845, 1.2)
							945.5421[M–H–(Ara(p)–H_2_O)]^−^ (945.5423, −0.2)
							915.5345[M–H–(Glc–H_2_O)]^−^ (915.5317, 3.1)
							783.4895[M–H–(Ara(p)–H_2_O)–(Glc–H_2_O)]^−^ (783.4895, 0.0)
							621.4370[M–H–(Ara(p)–H_2_O)–2(Glc–H_2_O)]^−^ (621.4366, 0.6)
16	F_1_	11.34	683.44[M–H+HCOOH]^−^	0.39–12.50	y = 694.40x − 181.40	0.998	683.4367[M–H+HCOOH]^−^ (683.4370, −0.4)
							673.4099[M+Cl]^−^ (673.4082, 2.5)
							637.4331[M–H]^−^ (637.4316, 2.4)
							475.3780[M–H–(Glc–H2O)]^−^ (437.3787, −1.5)
17	Rb_3_	11.38	1123.59[M–H+HCOOH]^−^	0.39–100.00	y = 276.60x − 71.21	0.999	1123.5892[M–H+HCOOH]^−^ (1123.5900, −0.7)
							1113.5605[M+Cl]^−^ (1123.5612, −0.6)
							1077.5856[M–H]^−^ (1077.5845, 1.0)
							945.5408[M–H–(Xyl–H2O)]^−^ (945.5423, −1.6)
							915.5325[M–H–(Glc–H2O)]^−^ (915.5317, 0.9^)^
							783.4913[M–H–(Xyl–H2O)–(Glc–H2O)]^−^ (783.4895, 2.3)
							621.4338[M–H–(Xyl–H2O)–2(Glc–H2O)]^−^ (621.4366, −4.5)
18	Rd	12.94	991.55[M–H+HCOOH]^−^	0.39–50.00	y = 92.99x + 42.17	0.997	991.5477[M–H+HCOOH]^− ^(991.5478, −0.1)
							981.5195[M+Cl]^− ^(991.5190, 0.5)
							945.5424[M–H]^−^ (945.5423, 0.1)
							783.4913[M–H–(Glc–H2O)]^−^ (783.4895, 2.3)
							621.4385[M–H–2(Glc–H2O)]^−^ (621.4366, 3.1)
19	F_2_	15.92	829.50[M–H+HCOOH]^−^	0.39–6.25	y = 615.80x − 143.40	0.998	829.4949[M–H+HCOOH]^−^ (829.4949, 0.0)
							819.4653[M+Cl]^−^ (819.4661, −1.0)
							783.4910[M–H]^−^ (783.4895, 1.9)
							621.4377[M–H–(Glc–H2O)]^−^ (621.4366, 1.8)
							459.3843[M–H–2(Glc–H2O)]^−^ (459.3838, −1.1)
20	Note-Ft_1_	15.93	961.53[M–H+HCOOH]^−^	0.39–6.25	y = 192.90x − 52.76	0.996	961.5366[M–H+HCOOH]^−^ (961.5372, −0.6)
							951.5073[M+Cl]^−^ (951.5084, −1.2)
							915.5300[M–H]^−^ (915.5317, −1.9)
							783.4902[M–H–(Xyl–H2O)]^−^ (783.4895, 0.9)
							621.4374[M–H–(Xyl–H2O)–(Glc–H2O)]^−^ (621.4366, 1.3)
							459.3856 [M–H–(Xyl–H2O)–2(Glc–H2O)]^−^ (459.3838, 3.9)
21	20(*S*)-Rg_3_	16.13	829.50[M–H+HCOOH]^−^	0.78-12.50	y = 553.50x − 202.10	0.999	829.4944[M–H+HCOOH]^−^ (829.4949, −0.6)
							819.4640[M+Cl]^−^ (819.4661, −2.6)
							783.4891[M–H]^−^ (783.4895, −0.5)
							621.4373[M–H–(Glc–H2O)]^−^ (621.4366, 1.1)
							459.3837[M–H–2(Glc–H2O)]^−^ (459.3838, −0.2)
22	*S*-PPT	16.51	521.38[M–H+HCOOH]^−^	0.39–200.00	y = 104.40x − 140.70	0.997	521.3850[M–H+HCOOH]^−^ (521.3842, 1.5)
							511.3556[M+Cl]^−^ (511.3554, 0.4)
							475.3803[M–H]^−^ (475.3787, 3.4)
23	PT	17.55	477.39[M+H]^+^	0.39–12.50	y = 686.70x − 90.04	0.999	477.3945[M+H]^+^ (477.3944, 0.2)
							459.3837[M+H–H_2_O]^+^ (459.3838, −0.2)
							423.3628[M+H–3H_2_O]^+^ (423.3627, 0.2)
							405.3518[M+H–4H_2_O]^+^ (405.3521, −0.7)
24	CK	18.02	667.44[M–H+HCOOH]^−^	0.39–12.50	y = 837.10x − 236.30	0.998	667.4426[M–H+HCOOH]^−^ (667.4421, 0.7)
							657.4142[M+Cl]^−^ (657.413, 1.4)
							621.4365[M–H]^−^ (621.4366, −0.2)
							459.3839[M–H–(Glc–H2O)]^−^ (459.3838, 0.2)
25	20(*S*)-Rh_2_	18.32	667.44[M–H+HCOOH]^−^	0.39–50.00	y = 419.50x + 167.20	0.996	667.4413[M–H+HCOOH]^−^ (667.4421, −1.2)
							657.4120[M+Cl]^−^ (657.4133, −2.0)
							621.4389[M–H]^−^ (621.4366, 3.7)
							459.3836 [M–H–(Glc–H2O)]^−^ (459.3838, −0.4)
26	20(*R*)-Rh_2_	18.56	667.44[M–H+HCOOH]^−^	0.39–12.50	y = 711.70x − 216.80	0.998	667.4426[M–H+HCOOH]^−^ (667.4421, 0.7)
							657.4121[M+Cl]^−^ (651.4133, −1.8)
							621.4384[M–H]^−^ (621.4366, 2.9)
							459.3822 [M–H–(Glc–H2O)]^−^ (459.3838, −3.5)
27	Rh_3_	23.08	649.43[M–H+HCOOH]^−^	0.39–200.00	y = 591.00x − 1246.00	0.998	649.4304[M–H+HCOOH]^−^ (649.4316, −1.8)
							639.4003[M+Cl]^−^ (639.4028, −3.9)
							603.4252 [M–H]^−^ (603.4261, −1.5)
28	*S*-PPD	24.26	425.38[M+H-2H_2_O]^+^	0.39–12.50	y = 257.30x − 8.87	0.997	499.3561[M+K]^+^ (499.3554, 1.4)
							483.3816[M+Na]^+^ (483.3814, 0.4)
							443.3890[M+H–H_2_O]^+^ (443.3889, 0.2)
							425.3788 [M+H–2H_2_O]^+^ (425.3783, 1.2)
29	*R*-PPD	24.42	425.38[M+H-2H_2_O]^+^	0.39–12.50	y = 160.00x − 20.73	0.999	499.3568[M+K]^+^ (499.3554, 2.8)
							483.3817[M+Na]^+^ (483.3814, 0.6)
							443.3890[M+H–H_2_O]^+^ (443.3889, 0.2)
							425.3785 [M+H–2H_2_O]^+^ (425.3783, 0.5)
30	PD	25.24	461.40[M+H]^+^	0.39–12.50	y = 396.30x − 39.50	0.999	483.3822[M+Na]^+^ (483.3814, 1.7)
							461.3400[M+H]^+^ (461.3995, 1.1)
							443.3892[M+H–H_2_O]^+^ (443.4889, 0.7)
							425.3787[M+H–2H_2_O]^+^ (425.3783, 0.9)
							407.3679[M+H–3H_2_O]^+^ (407.3678, 0.2)

* Mean measured mass (Da), theoretical mass (Da), mass accuracy (ppm).

**Table 2:** Sensitivity, precision, accuracy and stability of the assay.

No.	Analyte	LOQ (ng)	LOD (ng)	Precision (RSD, %, *n* = 6)		Spike Recovery (%, *n* = 3)			Stability (RSD, %, *n* = 6)
				Intra-day	Inter-day	Low	Middle	High	
**1**	Re	0.02	0.01	3.12	4.74	36.05	37.72	37.34	3.67
**2**	Rg_1_	0.02	0.01	3.05	4.42	44.87	45.79	44.51	2.48
**3**	Rf	0.05	0.02	4.13	3.60	105.31	94.45	99.28	2.70
**4**	Pseudo-F_11_	0.05	0.02	1.13	1.97	91.37	88.27	87.84	1.53
**5**	Pseudo-Rt_5_	0.02	0.01	4.66	5.21	114.14	114.88	113.79	4.16
**6**	*R*-Note-R_2_	0.02	0.01	2.15	3.33	111.52	111.80	103.19	2.32
**7**	20(*S*)-Rg_2_	0.02	0.01	2.21	4.27	102.75	100.13	109.37	3.38
**8**	20(*S*)-Rh_1_	0.02	0.01	2.52	3.60	101.41	105.32	108.20	2.17
**9**	20(*R*)-Rg_2_	0.02	0.01	2.04	3.86	105.39	107.03	108.61	2.11
**10**	F_3_	0.02	0.01	3.33	3.19	94.49	86.30	96.53	2.69
**11**	Rb_1_	0.02	0.01	1.78	2.97	114.84	114.42	114.06	1.37
**12**	20(*R*)-Rh_1_	0.02	0.01	2.28	4.18	91.11	90.49	96.39	2.74
**13**	Ro	0.02	0.01	2.17	3.80	93.49	86.41	91.03	2.11
**14**	Rc	0.02	0.01	2.68	3.92	90.16	89.62	92.76	1.85
**15**	Rb_2_	0.02	0.01	1.86	3.62	98.68	94.80	99.22	1.60
**16**	F_1_	0.02	0.01	2.41	3.20	105.00	95.98	94.01	2.17
**17**	Rb_3_	0.02	0.01	2.51	4.43	92.21	101.89	105.07	4.21
**18**	Rd	0.02	0.01	1.05	3.31	96.19	88.81	94.04	1.07
**19**	F_2_	0.02	0.01	5.18	4.71	95.86	93.77	97.74	6.13
**20**	Note-Ft_1_	0.05	0.02	7.27	5.90	105.65	106.86	109.60	7.73
**21**	20(*S*)-Rg_3_	0.02	0.01	6.17	4.58	95.60	107.17	100.73	6.37
**22**	*S*-PPT	0.05	0.02	4.49	3.98	100.42	98.45	98.60	3.31
**23**	PPT	0.05	0.02	1.21	2.38	88.08	113.21	114.41	1.82
**24**	CK	0.02	0.01	2.17	3.06	95.99	96.50	107.30	1.95
**25**	20(*S*)-Rh_2_	0.02	0.01	3.88	2.99	88.94	97.14	98.71	3.37
**26**	20(*R*)-Rh_2_	0.02	0.01	1.82	2.21	87.21	87.90	86.76	3.38
**27**	Rh_3_	0.02	0.01	1.12	2.14	109.27	96.83	93.76	1.24
**28**	*S*-PPD	0.05	0.02	4.82	4.41	90.75	112.73	113.56	2.58
**29**	*R*-PPD	0.05	0.02	8.79	9.46	96.37	91.49	91.58	3.91
**30**	PD	0.05	0.02	2.71	4.69	91.21	93.42	95.21	3.08

### 2.5. Quantitative Evaluation of Du-Shen-Tang and the Raw Materials

The newly established method was applied for the quantitative evaluation of Du-Shen-Tang preparations and the respective raw materials derived from ASG and AMG. Typical base peak intensity (BPI) chromatograms of methanol extracts and decoctions of ASG and AMG monitored in both negative and positive modes were shown in Figure 2B–D. The contents of all analytes interested were summarized in Table 3.

**Table 3:** Contents of 30 analytes in methanol extracts and decoctions of ASG and AMG samples (μg/g, *n* = 3)

No.	Analyte		ASG			AMG		
			Methanol Extract	Decoction	*P* ^b^	Methanol Extract	Decoction	*P* ^b^
**1**	Re ^a^	Ginsenosides with relatively higher polarity	3989.38 ± 1.29	3125.04 ± 1.56	0.035	12301.21 ± 4.49	12055.60 ± 4.49	0.004
**2**	Rg_1_^a^		8154.96 ± 2.93	7439.38 ± 4.62	0.003	713.49 ± 0.37	691.29 ± 0.44	0
**3**	Rf		1072.51 ± 2.14	883.59 ± 0.62	0.01	ND	ND	-
**4**	Pseudo-F_11_		ND	ND	-	3823.27 ± 5.95	3169.70 ± 2.40	0.012
**6**	*R*-Note-R_2_		ND	ND	-	ND	ND	-
**13**	Ro		3821.05 ± 3.72	1970.12 ± 0.99	0	1967.94 ± 0.55	1798.06 ± 1.30	0.005
**11**	Rb_1_		3368.43 ± 1.81	2328.44 ± 3.38	0	6950.76 ± 3.34	6766.78 ± 3.73	0.014
**14**	Rc		1521.44 ± 2.56	989.80 ± 2.04	0	2976.80 ± 6.23	2860.60 ± 6.20	0
**15**	Rb_2_		1399.51 ± 1.78	945.10 ± 1.87	0	346.25 ± 0.24	378.31 ± 0.48	0.004
**17**	Rb_3_		160.92 ± 0.17	128.86 ± 0.26	0.005	961.18 ± 0.51	737.80 ± 1.30	0
**18**	Rd		2564.89 ± 2.68	1582.96 ± 2.87	0	14986.82 ± 35.12	13407.25 ± 17.34	0
Total			26053.09	19393.29	-	45027.72	41865.39	-
**5**	Pseudo-Rt_5_	Ginsenosides with relatively lower polarity	ND	ND	-	14.28 ± 0.02	14.66 ± 0.03	0.001
**7**	20(*S*)-Rg_2_		93.50 ± 0.22	127.24 ± 0.17	0	222.41 ± 0.31	280.57 ± 0.47	0
**8**	20(*S*)-Rh_1_		24.09 ± 0.04	112.22 ± 0.33	0	ND	18.78 ± 0.05	0
**9**	20(*R*)-Rg_2_		ND	18.59 ± 0.03	0	5.34 ± 0.00	68.01 ± 0.05	0
**10**	F_3_		ND	ND	-	ND	ND	-
**12**	20(*R*)-Rh_1_		ND	54.98 ± 0.08	0	ND	BLOQ	-
**16**	F_1_		12.81 ± 0.00	13.39 ± 0.01	0.001	20.02 ± 0.03	19.37 ± 0.04	0
**19**	F_2_		11.23 ± 0.01	12.92 ± 0.01	0	334.25 ± 0.34	550.33 ± 1.10	0
**20**	Note-Ft_1_		ND	ND	-	ND	ND	-
**21**	20(*S*)-Rg_3_		18.09 ± 0.01	227.28 ± 0.15	0	27.31 ± 0.03	140.64 ± 0.06	0
**22**	*S*-PPT		ND	ND	-	BLOQ	BLOQ	-
**23**	PT		ND	ND	-	ND	ND	-
**24**	CK		ND	ND	-	49.53 ± 0.08	291.09 ± 0.57	0
**25**	20(*S*)-Rh_2_		ND	ND	-	ND	7.96 ± 0.01	0.001
**26**	20(*R*)-Rh_2_		ND	ND	-	ND	15.34 ± 0.01	0
**27**	Rh_3_		ND	ND	-	ND	87.29 ± 0.03	0
**28**	*S*-PPD		ND	ND	-	3.40 ± 0.02	8.22 ± 0.01	0
**29**	*R*-PPD		ND	ND	-	BLOQ	BLOQ	-
**30**	PD		ND	ND	-	ND	ND	-
Total			159.72	556.62	-	676.54	1502.26	-
Proportion of relatively lower to relatively higher polar ginsenosides			0.61%	2.87%	-	1.50%	3.59%	-

^a^ the contents = values calculated from calibration curves/average recovery. ^b^ *P* value of the T-test. ND: not detected. BLOQ: below the LOQ.

T-test for the content comparison of each investigated ginsenoside component between Du-Shen-Tang and its raw materials was performed. From Table 3, it could be found that significant differences (*p*< 0.05) in the contents of analytes interested were demonstrated between decoctions and methanol extracts of two ginseng samples (Table 3). In general, the contents of ginsenosides with relatively higher polarity (such as Re, Rg_1_, Rf, pseudoginsenoside F_11_, Rb_1_, Rb_2_, Rb_3_, Rc, Rd and Ro, *etc.*) were higher in the methanol extracts than those in the decoctions, whereas the contents of less polar ginsenosides (such as pseudoginsenoside Rt_5_, 20(*S*)-Rg_2_, 20(*R*)-Rg_2_, 20(*S*)-Rh_1_, 20(*R*)-Rh_1_, F_1_, F_2_, 20(*S*)-Rg_3_, *S*-PPT, CK, 20(*S*)-Rh_2_, 20(*R*)-Rh_2_, Rh_3_ and *S*-PPD, *etc.*) were higher in the decoctions than those in the methanol extracts. For example, the average total amount of higher polar ginsenosides decreased from 26053.09 μg/g in the methanol extract of ASG to 19393.29 μg/g in the respective decoction, whereas that of less polar ginsenosides increased from 159.72 μg/g in the same methanol extract to 685.37 μg/g in the ASG decoction. Similarly, the average total amount of relatively higher polar ginsenosides decreased from 45027.72 μg/g in the methanol extract to 41865.39 μg/g in the decoction of AMG, whereas that of relatively less polar ginsenosides increased from 676.54 μg/g in the methanol extract to 1502.26 μg/g in the decoction of the herb. Correspondingly, the proportion of less polar ginsenosides to those higher polar ginsenosides increased from 0.61% and 1.50% in the methanol extracts, to 3.53% and 3.59% in the decoctions, of ASG and AMG, respectively. All these results indicated that decocting could lead to altered proportion of less polar to higher polar ginsenosides in Du-Shen-Tang. As accumulated studies demonstrated that the bioactivities of less polar ginsenosides or aglycones are more potent, and their bioavailabilities are generally higher than those of the higher polar ones [8,9,10,11,12], the increased proportion of lower polar ginsenosides in ginseng decoction might be responsible for the “emergency treatment” efficacy of Du-Shen-Tang in TCM practice. Therefore, whether these altered proportion of different polar ginsenosides between ginseng methanol extracts and their decoctions will influence their bioactivities warrant further investigation.

## 3. Experimental

### 3.1. Chemicals, Reference Compounds and Samples

HPLC grade acetonitrile and methanol were purchased from Merck Co., Inc. (Darmstadt, Germany). MS-grade formic acid was supplied by ROE Scientific INC Co. (Dover, DE, USA). Ultra-pure water was produced by a Milli-Q water purification system (Milford, MA, USA).

Ginsenoside Re (**1**), Rg_1_ (**2**), Rf (**3**), pseudoginsenoside F_11_ (Pseudo-F_11_) (**4**), pseudoginsenoside Rt_5_ (Pseudo-Rt_5_) (**5**), 20(*R*)-noteginsenoside R_2_ (*R*-Note-R_2_) (**6**), 20(*S*)-Rg_2_ (**7**), 20(*S*)-Rh_1_ (**8**), 20(*R*)-Rg_2_ (**9**), F_3_ (**10**), Rb_1_ (**11**), 20(*R*)-Rh_1_ (**12**), Ro (**13**), Rc (**14**), Rb_2_ (**15**), F_1_ (**16**), Rb_3_ (**17**), Rd (**18**), F_2_ (**19**), noteginsenoside Ft_1_ (Note-Ft_1_) (**20**), 20(*S*)-Rg_3_ (**21**), 20(*S*)-protopanaxatriol (*S*-PPT) (**22**), panaxatriol (PT) (**23**), Compound K (CK) (**24**), 20(*S*)-Rh_2_ (**25**), 20(*R*)-Rh_2_ (**26**), Rh_3_ (**27**), 20(*S*)-protopanaxadiol (*S*-PPD) (**28**), 20(*R*)-protopanaxadiol (*R*-PPD) (**29**) and panaxadiol (PD) (**30**) were purchased from Chengdu Munster Biotechnology Co. Ltd. (Chengdu, China), and their structures are shown in Figure 1. The purity of these ginsenosides was higher than 95.0% by HPLC analysis.

Asian ginseng (ASG, JSPACM-03-65-1, 4 years old) and American ginseng (AMG, JSPACM-12-1, 4 years old) were collected from Tonghua and Fushun counties, Jilin Province in China on Nov. 2012. All herb samples were authenticated by Song-Lin Li from Jiangsu Province Academy of Traditional Chinese Medicine according to the monographs on Asian ginseng and American ginseng documented in China Pharmacopoeia (Part I, 2010 Version). The voucher specimens were deposited at Department of Pharmaceutical Analysis and Metabolomics, Jiangsu Province Academy of Traditional Chinese Medicine, Nanjing, China.

### 3.2. Sample Preparation

#### 3.2.1. Reference Compounds Solutions

Stock solutions: known amounts of ginsenoside Re, Rg_1_, Rf, 20(*S*)-Rg_2_, 20(*R*)-Rg_2_, 20(*S*)-Rg_3_, 20(*S*)-Rh_1_, 20(*R*)-Rh_1_, 20(*S*)-Rh_2_, 20(*R*)-Rh_2_, Rh_3_, Rb_1_, Rb_2_, Rb_3_, Rc, Rd, Ro, F_1_, F_2_, F_3_, *R*-Note-R_2_, Note-Ft_1_, Pseudo-F_11_, Pseudo-Rt_5_, CK, *S*-PPT, PT, *S*-PPD, *R*-PPD and PD were dissolved with methanol to get 30 stock solutions (about 1.0 mg/mL), respectively, and were stored under 4 °C. 

Reference compounds mixture solution: 200 μL of above 30 stock solutions were mixed and then reconstructed with methanol to get reference compounds mixture stock solution (final concentration of each compound is about 200 μg/mL). The mixed stock solution was serially diluted (dilution factor = 1, 2, 4, 8, 16, 32, 64, 128, 256, 512, 1024, 2048, 4096) to prepare working solutions of reference compounds. The working solutions were filtered by a 0.22 μm PTFE syringe filter before subjected to UHPLC-QTOF-MS/MS analysis.

#### 3.2.2. Ginseng Sample Solutions

Methanol extracts: 0.25 g of ginseng slices (ASG or AMG) was accurately weighed and ultrasonic-extracted with 10 mL of methanol for 60 min at room temperature. The extracts were filtered by a 0.22 μm PTFE syringe filter, and subjected to UHPLC-QTOF-MS/MS analysis. The samples were prepared and analyzed in triplicate.

Decoction samples: 0.25 g of ginseng slices (ASG or AMG) was accurately weighed and refluxed with 10 mL of water at 100 °C for 40 min. The extracts were rotary evaporated to syrup at 50 °C, and ultrasonic-extracted with 10 mL of methanol for 60 min at room temperature. The extracts were then processed and analyzed in the same manner as methanol extracts.

### 3.3. Ultra High Performance Liquid Chromatography (UHPLC)

UHPLC was performed on a Waters ACQUITY UPLC™ system (Waters Corporation, Milford, MA, USA) equipped with a binary solvent delivery system, auto-sampler, and a PDA detector. The chromatography was performed with a Waters ACQUITY HSS T3 (2.1 mm × 100 mm, I.D., 1.8 μm). The mobile phase consisted of (A) 0.1% formic acid in water and (B) acetonitrile containing 0.1% formic acid. The elution condition was optimized as follows: 5%–15% B (0–1 min), 15%–30% B (1–5 min), 30%–38% B (5–15 min), 38%–60% B (15–15.5 min), 60% B (15.5–23 min), 60%–95% B (23–23.5 min), 95% B (23.5–25 min), 95%–5% B (25–25.5 min) and isocratic at 5% B (25.5–27 min). The flow rate was 0.5 mL/min. The column and auto-sampler were maintained at 35 °C and 10 °C, respectively. The injection volumes of reference compound mixture solutions and sample solutions were 1 μL.

### 3.4. Mass Spectrometry

Mass spectrometry was performed on a Waters SYNAPT G2-S Q-TOF system (Waters MS Technologies, Manchester, UK) coupled with electrospray ionization (ESI) interface. The mass spectrometer was operated in both negative and positive ion modes. The desolvation gas was set to 900 L/h at 450 °C. The cone gas was 40 L/h. The source temperature was 100 °C. The capillary voltage and cone voltage were set at 2500 V and 30 V, respectively. The Q-TOF acquisition rate was 0.2 s. The energies for collision-induced dissociation (CID) were 6 V for the precursor ion and 30–60 V for fragmentation information. The mass accuracy and reproducibility were maintained using a LockSpray™. The [M−H]^−^ (*m/z* 554.2615) and [M−H]^+^ (*m/z* 556.2771) ions of leucine-enkephalin (1 ng/μL infused at 5 μL/min) were used as reference lock mass. Centroided data were acquired for each sample over a mass range of *m/z* 100–1500 with dynamic range enhancement (DRE™) applied throughout the MS experiment to ensure accurate mass measurements. The accurate mass and elemental composition for the precursor ions and the fragment ions were calculated using the MassLynx V4.1 software (Waters Co., Milford, MA, USA).

### 3.5. Method Validation

#### 3.5.1. Linearity and Sensitivity

The linearity and sensitivity of the method were evaluated by calibration curves, limits of detection (LOD) and quantification (LOQ), respectively. Working solutions of the mixed reference compounds were diluted to appropriate concentrations for the construction of calibration curves. Thirteen different concentrations of each compound were analyzed in triplicates, and then the calibration curves were constructed by plotting the peak areas of each extracting ion *versus* the concentrations of respective analyte.

The stock solutions of all reference compounds were serially diluted with methanol, and an aliquot of the diluted solutions were subjected to UHPLC-QTOF-MS/MS analysis. The LODs and LOQs under the aforementioned analytical conditions were determined at an S/N (signal to noise) of 3 and 10, respectively. The values of LOD and LOQ were calculated through the concentrations of mixed reference multiplied by injection volume (1 μL).

#### 3.5.2. Accuracy, Precision and Stability

The spike recovery test was conducted to evaluate the accuracy of the method. The ASG and AMG samples were mixed at equal amounts. The herbal mixture was then extracted with methanol and analyzed in triplicate to determine the original amount of each analyte in herbal mixture. Then different amounts (high, middle and low level, calculated by 120%, 100% and 80% of original contents in the herbal mixture, respectively) of the 30 analytes were weighted and spiked into the herbal mixtures. The resultant mixtures were then extracted with methanol and analyzed in triplicates to determine total amount of each analyte. The spike recoveries were calculated from the following equation:

Spike recovery (%) = (total amount detected-amount original)/amount spiked × 100%

Intra- and inter-day variations were obtained to assess the precision of the developed assay. For intra-day variability test, the equal amounts of ASG and AMG samples were mixed (totally 0.25 g) and then spiked with middle concentration level (100% of original contents in the herbal mixture) of 30 reference compounds. The resultant mixtures were extracted with methanol and analyzed in six replicates within one day. In inter-day variability tests, the same sample was analyzed in duplicates for three consecutive days. The relative standard deviation (R.S.D.) was taken as a measure of precision

The stability test was performed by analyzing the samples prepared for the precision test at appropriate time intervals (0 h, 2 h, 4 h, 6 h, 8 h, 10 h, 12 h, 16 h, 24 h). The RSDs of the peak areas of each analyte were taken as the measures of stability.

## 4. Conclusions

In present study, an UHPLC-QTOF-MS/MS method for simultaneous determination of 30 ginsenosides and relevant aglycones was developed and validated, and successfully applied to evaluate the holistic quality of Du-Shen-Tang, the decoction of ginseng. Compared with the published LC-QTOF-MS method for quantification of ginsenosides, the newly established one could separate more analytes (30 *vs.* 15) within less time (26 min *vs.* 35 min). By adopting the step wave ion-transfer optics technology of QTOF-MS, the quantification limit of ginsenosides interested reached less than 0.05 ng. Furthermore, with the accurate mass measurement and wide linear range of QTOF-MS/MS, simultaneous qualification and quantification of these 30 analytes were obtained. Significant changes in the contents of different polar ginsenosides and aglycones in Du-Shen-Tang derived from ASG or AMG were observed. The results suggested that this newly developed method is fast, selective, sensitive, precise and accurate enough for the quality evaluation of Du-Shen-Tang, and may also be applied to the extensive investigation of ginseng and its related preparations.
